# Ten simple rules for improving research data discovery

**DOI:** 10.1371/journal.pcbi.1009768

**Published:** 2022-02-10

**Authors:** Nicole Contaxis, Jason Clark, Anthony Dellureficio, Sara Gonzales, Sara Mannheimer, Peter R. Oxley, Melissa A. Ratajeski, Alisa Surkis, Amy M. Yarnell, Michelle Yee, Kristi Holmes

**Affiliations:** 1 NYU Health Sciences Library, NYU Langone Health, New York, New York, United States of America; 2 Montana State University Library, Montana State University, Bozeman, Montana, University States of America; 3 Medical library, Memorial Sloan Kettering Cancer Center, New York, New York, United States of America; 4 Galter Health Sciences Library and Learning Center, Northwestern University Feinberg School of Medicine, Chicago, Illinois, United States of America; 5 Samuel J. Wood Library and C.V. Starr Biomedical Information Center, Weill-Cornell Medicine, New York, New York, United States of America; 6 Health Sciences Library System, University of Pittsburgh, Pittsburgh, Pennsylvania, United States of America; 7 Health Sciences and Human Services Library, University of Maryland—Baltimore, Baltimore, Maryland, United States of America; 8 Department of Preventive Medicine, Northwestern University Feinberg School of Medicine, Chicago, Illinois, United States of America; Dassault Systemes BIOVIA, UNITED STATES

## Introduction

Sharing and reusing research data present both opportunities and challenges for the individual researcher, their organizations, and the entire research community, but the promise of data sharing can only be actualized if the right data can be found. While grants and publications increasingly include data sharing requirements, locating the right data to answer a research question can still be challenging. As more data is shared more frequently, the data discovery problem becomes more apparent. There is simply more data to look through, and that data is distributed across a growing number of repositories, article supplements, websites, and other locations with different metadata, data standards, and search functionality.

These 10 rules can be thought of as a mirror to “Eleven Quick Tips for Finding Research Data,” providing key guidance on how to make your research data more findable in the complicated systems that share and provide access to research [1]. As opposed to helping locate data for reuse, this article is meant to help you make your data and your research more discoverable. The rules below walk you through the process of making your data more discoverable, including key steps to take when publishing an article (Rules 8 and 9).

As members of the Data Discovery Collaboration (DDC) [2], our work focuses on the issue of data discovery. We are a community of librarians and information professionals and are invested in helping to improve data findability and open data infrastructure more broadly. In service of these goals, we created these rules to provide guidance on how to improve the discovery of your research data by making key decisions early in the process (Rules 1 and 2), leveraging scholarly infrastructure already in place (Rules 3, 4, 5, 6, and 7), taking important steps at the point of publication (Rules 8 and 9), and tapping into the growing community of data professionals (Rule 10).

## Rule 1: Decide what level of access you can provide

Data discovery is not data access. You can make your data discoverable without making it openly available. Data discovery is the process of learning that a dataset exists, whereas data access is the ability to download or view that data. Both of these concepts are part of the FAIR Data Principles (Findable, Accessible, Interoperable, Reusable) [3], guidelines for data sharing that have been adopted by the National Institutes of Health (NIH) and many others. Discovery, the “F” (Findable) in FAIR, and access, the “A” (Accessible), are both integral concepts in improving data sharing.

That said, depending on data privacy issues, as well as security and ethical considerations, there may be restrictions placed on data access, such as an application to the data owner or International Review Board (IRB) or Ethics Committee approval. Deciding the level of access that you can provide to data will impact how you describe your data and what tools you use to make your data discoverable. As such, deciding the level of access you can provide to a dataset is the first step in making your data discoverable.

The distinction between data discovery and data access is reflected in the tools used to explore research data, such as data catalogs and repositories. A data catalog is a tool that holds metadata that explains the who, what, when, where, and why of a dataset [4–7]. Metadata is searchable information, formally defined as “…structured information that describes, explains, locates or otherwise makes it easier to retrieve, use or manage that resource” [8]. A data repository is a tool that holds that metadata along with the data itself [9]. For example, the UK Data Service’s Data Catalogue [10] describes data and where it is stored, while ReShare, a repository [11], stores data and employs metadata to help users locate the right dataset within their own storage.

Consider optimizing the discovery of restricted data through a data catalog, particularly since there may be limited options for repositories that can accommodate restricted data. Data catalogs require only a description of the data and access procedures, and thus can make the existence of the data and the processes for potentially gaining access to it transparent, without violating restrictions on accessing the data itself. Your first step in improving the discovery of your research data is to know what access restrictions you may be subject to and how much information can be shared with the public.

## Rule 2: Comply with ethical standards

As you decide whether you can provide access to your data and if you can make your data discoverable for others to apply for access, you will need to comply with a range of ethical regulations. Ethical considerations for data should come into play across all stages of a project—not only in the generation of data, but also in sharing, discovery, and reuse [12]. Regulations provide some direction when considering ethical practices for data discovery, but ethics should be considered as an active discussion over the course of a project in order to accomplish the work in accordance with your values as well as in compliance with regulations in your country and in your field. Your values and these regulations should be used to inform how data is used, stored, and shared during the life of the project. Explicitly defining acceptable procedures and practices with your data and basing it in your ethical values can ensure good and ethical data management across all stages of a project [13].

Relevant regulations vary internationally. The revised Common Rule [14], which went into effect in the United States in 2019, introduces guidelines for data reuse, including the idea of broad consent—that participants can consent to “future storage, maintenance, or research uses” of their data [15]. In the European Union, 2018’s General Data Protection Regulation (GDPR) provides some guidance regarding discovery and use of existing data; however, the GDPR’s ramifications for academic research are still not fully clear [16,17]. Understanding regulations relevant to your location and field as well as the guidelines of local regulatory bodies, like your IRB or Ethics Committee, can help guide decisions on whether to make your data discoverable and how. Below are some considerations to support ethical practice for data discovery, based on key elements of the research and clinical ethics, as outlined in the United States’ Belmont Report [18]:

1. Respect for persons (i.e., human dignity). Review your consent agreements and consider how you might make your data discoverable while respecting participant consent.
2. Beneficence (i.e., minimizing harm and maximizing benefit). What are the potential risks of making your data discoverable as weighed against the potential harms? If your data is discoverable, will it negatively affect human subjects, endangered species, protected lands, or others?
3. Justice (i.e., fair treatment and privacy). When working with human subjects data, carefully consider the risk of reidentification when setting access procedures or choosing tools to enhance discovery. Consider direct and indirect identifiers and any related datasets that could lead to reidentification. Also consider intellectual property and ownership—is the data yours to make discoverable?

If your data results from work with vulnerable populations, be particularly careful. If applicable, consider referring to the CARE Principles for Indigenous Data Governance (Collective benefit, Authority to control, Responsibility, Ethics). These principles, meant to complement the FAIR principles, provide key guidance on ways to work with indigenous individuals and communities and data that describes them [19]. Remember, supporting discovery for data does not mean that they need to be openly accessible; setting access procedures can allow for ethical data discovery and sharing [6,20]. Finally, it will be beneficial to consider your own values and ethical codes when pursuing newer practices like data publishing, discovery, and reuse, which may introduce new concerns not yet represented in regulations and guidelines [21–23].

## Rule 3: Deposit your data somewhere trusted

In some cases, deciding where to describe or store your data to make it discoverable is an easy decision. Sometimes funder will require use of a specific repository, or, in other cases, a repository will be the clear choice for a specific type of data. For example, the Gene Expression Omnibus (GEO) database and the database of Genotypes and Phenotypes (dbGaP) are clear choices for depositing gene expression data and genome-wide association study (GWAS) data, respectively, for high visibility. Both GEO and dbGaP are well known and widely used NIH resources for preserving and making these specific types of data available. Other researchers looking for gene expression data will know to go to GEO, making it discoverable to the relevant research community. For other types of data, the decision may be less clear as there may not be a repository with a similar degree of buy-in, longevity, and support.

When the choice is not as clear, how do you identify an appropriate home? For discovery purposes, it is generally best to look for a discipline-specific repository because fellow researchers with similar research aims are more likely to look in those repositories for data to reuse. A number of journals and publishers that require data sharing have evaluated repositories based on their stability and practices and provided a list of recommended repositories [24–26], offering a good place to start. For those working in the United States, the NIH maintains a list of NIH-supported repositories [27], and that support provides a degree of confidence in their sustainability. Included in that list are some repositories that enable restricted access, so if your data cannot be freely shared, a NIH-supported repository may still be an option.

Other discipline-specific repositories can be identified using the Registry of Research Data Repositories [28]. Keep in mind that repositories vary widely in their degree of buy-in from research communities and sustainability and support from institutions who fund them. When choosing a repository, consider whether the repository is sustainable. Will it still exist in 5 years? Are there signs that it may lose the resources to accept new data and thereby becomes overlooked, with nonoptimized records? While there is no crystal ball to predict which repositories will be around and active in the future, considering the length of existence, the user population, and funding sources may provide a sense of whether or not a repository is likely to stick around.

If there are no suitable discipline-specific repositories, the publisher and NIH lists also include generalist repositories [24–27] that accept data from any discipline. These repositories vary in their size limitations, costs, and other policies [29]. In cases where datasets would be of interest to people in a broad array of disciplines, a generalist repository may be the best choice for data discoverability.

Another option is to upload your data to an institutional repository. These locally maintained generalist repositories may be a particularly good option for sustainability since an institution has a vested interest in preserving its data assets; however, make sure that the deposited data is findable through search engines (e.g., Google Dataset Search) or dataset aggregators (e.g., Mendeley Data). In fact, regardless of the type of repository you choose, checking to see that it is included in dataset aggregators and search engines should be an important part of your decision. These bolster the discovery of data stored across the many repositories.

In the interest of open science, it is strongly recommended to deposit your data to a repository to support data access, preservation, and reuse. A repository will help ensure a sound storage and preservation strategy for your data [13], and as stated earlier, if you need to limit access to your dataset, there are some repositories that can provide restricted access options. However, sometimes this is not possible. In these situations, a “landing page” record in a data catalog or repository is recommended if that option is available to you. While not every institution has a data catalog or repository with this function, if yours does, this is an excellent option for making data discoverable while you, or perhaps a third party, maintain control over the data.

## Rule 4: Use persistent identifiers

Persistent identifiers (PIDs) are meant to provide a more stable, long-lasting way of uniquely identifying digital objects, people, and institutions and are vital for locating and citing scholarly materials [30]. Throughout the process of making your data discoverable, PIDs will play a crucial role. PIDs provide significant benefits: They can be machine readable, citable, and bound with metadata about the object, person, organization, or concept to which they point. Registry organizations maintain PIDs and provide search interfaces and APIs [31,32] for querying their registries. This means that by using PIDs and being registered through these organizations, you provide another avenue by which people may search for and discover your data. Assigning and using a PID can also makes it more possible to track the use and reuse of data over time, providing a clearer view on downstream use and dissemination.

To better explain PIDs and their benefits, we will highlight 2 important examples of PIDs: Digital Object Identifiers (DOIs) [33] to distinguish digital objects such as journal articles, and ORCID iDs [34] to distinguish between yourself and other researchers. ORCID iDs are identifiers for researchers and can help support scholarly identity management. These identifiers disambiguate you from other researchers with similar names, collapse variations of your own name to a single referent, and can maintain information about scholarly affiliations, education, funding, publications, and other scholarly works. ORCID iDs have been incorporated into publishing and funding workflows, resulting in a rich network of connections that can enhance the discovery of your data and other research products [35].

DOIs are used to distinguish digital research objects, including publications, data, software, and supporting information (see Rule 8). When a DOI is attached to a digital object, it is possible to accurately assert relationships between a given research object and other entities (e.g., authors with ORCID iDs) through links. Most publishers and repositories have incorporated DOIs, making these linkages, and thus enhanced discovery, possible. Furthermore, many repositories assign data a DOI upon deposit, making it easy to use and benefit from DOIs.

## Rule 5: Create thoughtful and rich metadata

As emphasized in the FAIR Data Principles, data should be findable (the “F” in FAIR) both by humans and machines [3]. This means that the data needs to be described using metadata in a way that humans can easily read as well as in a structured way that machines can parse and link to other resources. PIDs can greatly aid in this process, but to increase discoverability, you will also need to describe your data with rich metadata. As previous 10 Simple Rules papers have noted, making material findable and understandable for humans and for machines is key to ensuring that the materials themselves are as useful as possible [30,36]. As such, adding metadata is a good step forward in making things findable, but adding metadata that uses a structured metadata schema is crucial [37].

A metadata schema is a system that defines the data elements needed to describe a particular object, such as a certain type of research data. A metadata schema tailored to your discipline provides a set of metadata elements designed to provide a description of your dataset that is sufficient to make it discoverable and understandable. For example, the Content Standard for Digital Geospatial Metadata (CSDGM) is a metadata standard for geographic data, maintained by the Federal Geographic Data Committee [38], while Data Discovery Initiative (DDI) is used to describe social science data [39]. Whether depositing a dataset into a repository or describing it in a data catalog, there will most likely be required data elements to complete. This rule is focused on how best to address several key metadata elements that are common to nearly all repositories and data catalogs: title, contributors, description, and funding.

When individuals are searching for datasets to reuse, they most likely will use the title as the first criteria to determine if it meets their needs. Be descriptive with your title and avoid defaulting to the filename as the title. Consider including as many of the following as are relevant to your dataset: What (type of data), Where (was the data collected), When (timeframe of the data), Who (subject of the research), and the Scale (approximate size) of the dataset.

Next, include the name and ORCID iD of all persons who contributed to the dataset (see Rule 4). The list of people who contributed to the dataset may be different from those with authorship on a related publication. Contributor roles to consider include data curation, methodology, resources, and software [40].

A dataset description is usually the lengthiest portion of a metadata record and the one that requires the most thought. This description should not be a restatement of a publication’s abstract nor should it address any results, analysis, or conclusions, although it may briefly describe the original research question with contextual details. For example, a description might start: “This dataset was generated from a survey that studies…” or “This dataset includes sequencing data from…” It can include information about the number and types of files included, important variables, and other documentation available (e.g., survey instruments, README files, information on research software). If the repository or catalog you are using does not have specific metadata elements for important experimental elements such as species/strain, cell type, study population, equipment, or other details, work them into the description text. This text will generally be searchable and therefore aid in the dataset’s discoverability. Most importantly, while writing the description, think about how you yourself would go about trying to find a dataset like yours. What terms would you use to search for the dataset and what would be most important to know in order to determine if you could reuse it?

Finally, provide specifics about funding that supported the work that produced the dataset (e.g., such as the funding organization, program name, and grant number) and provide links to project information in tools like NIH RePORTER [41]. Including this information will credit the funding agency for supporting the creation of the dataset, and it will also allow others search for research connected to a grant to find your dataset more easily.

## Rule 6: Choose your keywords carefully

While a repository or data catalog may not require you to submit keywords, you can further enhance your dataset record’s discoverability by providing descriptive keywords and using controlled vocabularies or ontologies. Picking the right keywords is a vital part of the description of your data and is key to improving the discoverability of your data and other research outputs [30].

Controlled vocabularies and ontologies are resources maintained online by professional organizations and associations that standardize terms commonly used in various professional contexts, a common example of this being ICD-10 codes. These standardized terms are also machine readable, meaning that related keywords can be connected and enhance search results [36]. Medical Subject Headings (MeSH) [42], a hierarchically organized list of medical terms developed for PubMed, is one such example and is popular in medical data repositories. Some repositories offer ways to search for your keywords and related terms directly in these vocabularies through API integrations.

While using controlled vocabularies and ontologies is the best practice, data repositories do not always support their use. In which case, choosing keywords can increase the discoverability of your dataset by providing (1) the ability to describe your dataset with additional topical terms that might not be in the title or description fields; and (2) the opportunity to add synonyms, as well as broader or more granular terms that someone interested in the dataset might use in a search. Additionally, repositories and catalogs frequently allow users to browse all datasets with a specific keyword, meaning that adding keywords will also make your data discoverable through browsing functions.

An example of the benefits of tagging data records with synonyms can be seen in the generalist repository Zenodo, in which a search of “coronavirus” returns around 1,270 results at the time of writing whereas a search for the synonym “covid-19” returns around 58,700 results. In either set of records, a user can click on one of the hyperlinked keywords to do a targeted search for that keyword term. When adding keywords, whether or not they are part of controlled vocabularies, think of the words you would use to locate a similar dataset, then check whether these words are present in the description. If not, add them as keywords. Such terms might be related to geographic coverage, software used, instrumentation used, common abbreviations, subject of study or subject domain, to name only a few possibilities. A few minutes’ effort in identifying and applying synonym, variant, or more granular keyword terms can make all the difference to a searcher for data records and to your data’s overall discoverability and thus impact.

## Rule 7: Create links to related resources

Data, along with publications, software, and other research products, are part of the larger research ecosystem. The research ecosystem includes research objects (e.g., articles, datasets, associated files, data management plans, code), the people who create these objects, funders and organizations that provide support for those objects, etc., and even information on how these concepts are linked. Being intentional about how you link your dataset to other components in this research ecosystem is an essential step toward improving the discoverability of your data. For example, use of a Data Availability Statement (see Rule 9) links your data to your publication, yet, there are more things to link than just your publications and your datasets.

Common opportunities and spaces for creating linkages across this network include links from labs or personal websites, links on social media posts back to research objects, a news post that announces your research findings and links to the release of the dataset, a link to a lay summary describing those findings for the public, a link from a code repository, and linking your research objects within an established metadata record. When creating these links, it is a good idea to follow best practices for other types of research objects. For example, research groups like the FORCE11 Software Citation Working Group have created guidelines on citing software [43] that would help you provide persistent links between your data and your software.

With discovery as your goal, this last example is the best place to start. After placing your dataset in a repository, enhancing the metadata record for your dataset by linking it to the software used for data collection using the software citation principles can provide additional context to your dataset that simple metadata cannot provide. To get the most out of these linkages, you will also want to use PIDs, as described in Rule 4.

## Rule 8: Make supporting information discoverable too

These next 2 rules discuss ways to improve data discovery while in the publication process. When writing a manuscript and publishing, you will frequently create PDFs, CSV spreadsheets, or additional figures as supporting information. There are key differences between a data file submitted as a supporting information for a journal article versus a data file that has been preserved and shared in a repository. Supporting information added to journal articles are far less discoverable than files stored in repositories. Supporting information are linked to within the journal article and can be hosted by journals themselves. While it may be tempting to “set it and forget it” and solely deposit supporting information with a journal, these files may be overlooked (e.g., poorly located on the page or lack description), may be hidden behind journal paywalls, or become inaccessible if their links are not maintained [44–46].

Moreover, the naming of supporting information may be garbled, truncated, or replaced with a random sequence of characters, impacting long-term discoverability and usability. Think about how frequently supporting information are mysteriously named “cc9-12-e0123-s001.pdf” or “2020_1234_MOESM2_ESM.xls” and attached to an article in PubMed Central. If multiple such files are listed, it is impossible to know which file contains the desired data. Also, consider what could be missing. Due to costs associated with the storage and maintenance of supporting information, some journals impose limits on the size of deposited files [47], which means a submitter may not be able to deposit the full complement of files needed to support subsequent use.

Additionally, journals rarely commit to preserve supporting information in perpetuity. These and other factors necessitate finding an alternate hosting solution for data before submitting a manuscript to a journal. As mentioned in Rule 3, an important step in improving data discovery is depositing data somewhere trustworthy and persistent. Depositing these supporting information in other resources, like an institutional repository, helps ensure that access to these files will be maintained over time. Data files that are stored in repositories are indexed and maintained as separate entities. If best practices are followed (e.g., descriptive metadata and unique identifiers are applied), these files will appear in search engines on their own and are therefore opportunities to increase discoverability of a study and your visibility as a researcher.

The NIH Policy for Data Management and Sharing, set to take effect in 2023, encourages researchers to deposit high-value data files in related subject or data type–specific repositories, with generalist and institutional repositories and PubMed Central (for files under 2GB) as secondary options [48]. However, for all of the reasons noted above, the authors suggest utilizing repositories and linking them rather than hosting files solely within PubMed Central.

## Rule 9: Include an accurate Data Availability Statement with your publications

A Data Availability Statement (DAS) within a journal article provides detailed information on where the data backing the claims of the research are located, whether that data is available for review, and if it is not, why it is not available. Several journals have provided recommendations on the specific style, format, and content of a DAS [49,50]. Writing a DAS is an important part of publishing that improves the visibility of your research data.

Writing a true and persistent DAS can provide a host of benefits, including increasing the discoverability of your data. First, it allows you to link directly to your data from a publication. Second, if a dataset cannot be deposited in an indexed repository for ethical, regulatory, or legal reasons, a DAS increases the visibility of this data that may be otherwise undiscoverable. Third, a DAS allows you to leverage filters in literature databases, making your publication more visible to researchers looking for secondary datasets. For example, in PubMed Central, it is possible to search only for articles that include a DAS so including a DAS increases the odds that a researcher looking for data to reuse will locate your research. Furthermore, there is evidence to suggest that the inclusion of a DAS leads to a citation advantage for authors [51].

Analysis has demonstrated that researchers often fail to write an accurate and useful DAS, but creating one does not need to be a difficult process [52]. In the case where data has been shared through a repository, there are only 2 key elements to a DAS. These components are a description of where the data is stored (e.g., a repository) as well as information to identify the dataset within that storage facility (e.g., accession information, a DOI, or a permalink).

However, for datasets that are restricted due to subject privacy or other regulatory issues, a different type of DAS is necessary. In this case, it is customary to include a short explanation of why the dataset is restricted along with information on who to contact in order to apply for access to the confidential data. This approach can present a barrier to those interested in using a dataset due to the difficulty of locating the owner of the data and managing the access procedures. It can also present issues for data owners who are then required to perform additional work to share the data years after publication. For this reason, this method should only be employed if a dataset cannot be deposited into a repository. In the case where there is no other option, you should ask yourself the following critical questions:

- Is there a process for preparing this data for another researcher when it is requested?
- Is there sufficient documentation to allow others to understand the dataset?
- Is accurate and current contact information provided?
- Has there been a regular review of stored data?
- Is there a person assigned to act as a data steward and perform any necessary tasks when data is requested?

Sometimes a hybrid approach is best. A subset of a restricted dataset (e.g., a deidentified dataset) can be made available through a repository. This hybrid approach entails including access information for the accessible subset of the restricted dataset, information on why the larger dataset is restricted, and contact information to get further information on the larger dataset. Of course, before submitting a DAS, proofread the accession numbers and test any links to help ensure that the DAS is true, specific, and persistent.

## Rule 10: Talk to your institutional librarian

You are not in this alone. You have subject matter experts in the field of discoverability close at hand, ready to work with you to support practices in data sharing in order to enhance access, support discoverability, and respect privacy.

The NIH and National Science Foundation (NSF) data management policies recommend institutional librarians as sources of expertise on issues related to data discoverability [48,53,54]. Librarians work with metadata, repositories, ontologies, and data management plans from both sides—searching and curating—and are highly familiar with institutional, national, and international requirements and policies for data handling. Your institutional library can therefore provide guidance in best practices, teach you how to use available tools and templates, as well as help you select appropriate repositories and data catalogs to maximize the discoverability of your data. Check your library for consultation services or classes in data management or for research guides and other online resources available to you.

Working through data discoverability is like preheating your oven: It is best done at the beginning of the process to prevent unpleasant delays at the end. Being proactive will help confirm that your data management is aligned with any regulatory requirements at the beginning of your project, and may reveal workflows that will allow efficient metadata collection and sharing along the way.
